# Body composition analysis in adolescents idiopathic scoliosis and its correlation with the severity of scoliosis

**DOI:** 10.1038/s41598-025-12674-4

**Published:** 2025-07-26

**Authors:** Shilei Li, Hongqi Zhang, Qile Gao, Shaohua Liu, Yuxiang Wang

**Affiliations:** 1 Present Address: Department of Spine Surgery and Orthopedics, Xiangya Hospital, Central South University, No. 87, Xiangya Road, Changsha, 410008 Hunan China; 2 National Clinical Research Center for Geriatric Disorders, Xiangya Hospital, Central South University, Changsha, China

**Keywords:** Adolescents idiopathic scoliosis, Muscle percentage, Subcutaneous fat percentage, Body composition, Body fat scale, Medical research, Outcomes research

## Abstract

To analyze the body composition of adolescents idiopathic scoliosis(AIS) patients at different stages of skeletal maturity and to explore the correlation between body composition and the severity of scoliosis. Data were collected from 243 patients diagnosed with AIS who fulfilled the specified inclusion and exclusion criteria at the Spine Surgery Outpatient Clinic of Xiangya Hospital, Central South University, during the period from 2023 to 2024. The data encompassed basic demographic information, radiological assessments, and body composition metrics. Patients were categorized based on Risser grade (0–5) and Cobb angle, with classifications of mild (< 20°), moderate (20–40°), and severe (≥ 40°) scoliosis. A comparative analysis of body composition across the different groups was conducted, alongside an investigation into its correlation with the severity of scoliosis. As the Risser sign grade increases, the age, height, weight, and BMI of AIS patients gradually increase (*p* < 0.05), while the muscle percentage of the whole-body, trunk, and limbs gradually decrease (*p* < 0.05). Patients in the mild group have significantly higher muscle percentage and significantly lower subcutaneous fat percentage compared to the moderate and severe groups (*p* < 0.05) ; the whole-body muscle percentage and the ratio of it to subcutaneous fat percentage have a weak negative correlation with the rotation of the apical vertebra (AVR) (*r* = − 0.131, *p* = 0.042; *r* = − 0.169, *p* = 0.009), and the whole-body subcutaneous fat percentage has a weak positive correlation with AVR (*r* = 0.150, *p* = 0.021). In AIS patients, as skeletal maturity progresses, there is a gradual decrease in both whole-body and local muscle percentage. The whole-body muscle percentage and whole-body subcutaneous fat percentage exhibit a weak correlation with the curvature associated with scoliosis. During conservative treatment, increasing the percentage of whole-body muscle mass while reducing the percentage of whole-body subcutaneous fat may be beneficial for patients with AIS.

## Introduction

AIS is a condition that affects adolescents with an unknown etiology, with a prevalence of 1–3%, and a higher incidence in females than males. The etiology is complex and multifactorial, involving genetic factors, hormonal secretion, uneven growth of vertebral bodies, abnormal muscle activity, postural issues, or a combination of these factors^1–3^. Severe scoliosis not only affects physical health but also impacts mental well-being, making the study of its causes and the prevention of its progression of significant importance.

Previous studies^4–6^ have indicated that many scoliosis patients exhibit issues related to bone metabolism and abnormalities in the paraspinal muscles. Low bone mass and low body weight appear to be systemic phenomena in AIS, with patients having lower weight and bone mass experiencing more severe curvature^4–6^. The study conducted by Sun et al.^7^ demonstrates that reduced bone mass serves as an independent risk factor for the progression of spinal curvature in patients with adolescent idiopathic scoliosis (AIS) undergoing brace treatment. The authors advocate for the assessment of baseline bone mineral density (BMD) in AIS patients prior to the initiation of brace therapy to better predict treatment outcomes. Furthermore, animal studies have indicated that addressing osteopenia in mouse models of adolescent idiopathic scoliosis can lead to improvements in scoliosis^8^. Additionally, vitamin D supplementation is recommended to enhance bone mineral density in individuals with AIS^9^. Through research focused on the bone mass of patients with AIS, novel treatment methodologies have been developed for this population. However, there are relatively few studies on other body components of AIS patients. Consequently, recent research efforts have increasingly concentrated on the body components of AIS patients, with the objective of acquiring a more comprehensive understanding that may inform clinical interventions. A study comparing body composition between AIS patients and healthy controls found that reductions in total muscle mass and fat mass are associated with an increased risk of adolescent scoliosis^10^. Comparisons with normal controls have revealed that AIS patients are thinner due to both lower skeletal muscle mass and body fat^11^. Existing research on body composition in AIS has yielded conflicting results, a study by Ramirez et al.^12^ showed that the fat-free mass index in AIS was lower than in the normal control group, while a study using the Carter equations to estimate body composition in AIS girls indicated no difference in total body muscle mass between the AIS and normal control groups^13^. Some studies have also indicated^14^ that the total muscle mass in severe group is lower than that in moderate group. Muscle tissue is a vital organ, constituting a significant proportion of body weight, and skeletal muscle has a cross-regulatory effect on bones, there is a known association between muscle mass and bone density, with sarcopenia and osteoporosis often developing concurrently^15,16^. Consequently, variations in muscle mass may play a significant role in the development and progression of AIS. Back muscles play a crucial role in maintaining spinal balance^17^. Current research on the muscle tissue of patients with AIS primarily concentrates on the local paraspinous muscles, which include the erector spinae, multifidus, and lumbar muscles., and it has been confirmed that there are asymmetric changes on the concave and convex sides of the curve^18,19^. Furthermore, research conducted by Ohashi et al.^20^ indicates that the use of braces during adolescence in AIS patients does not adversely affect the development of trunk muscles. However, the relationship between trunk muscle mass and the severity of scoliosis remains unclear.

The investigation of body composition in patients with Adolescent Idiopathic Scoliosis (AIS) holds considerable significance. Previous studies predominantly focused on comparing the body composition of AIS patients with that of the general population, thereby elucidating the differences between these two groups. While such research has enhanced the understanding of body composition in AIS patients and offered new insights for clinical interventions, inconsistencies in the findings of various studies persist. Consequently, there is a pressing need for further research to delineate the characteristics of body composition in individuals with AIS. Moreover, the alterations in body composition of AIS patients throughout their growth and development remain inadequately understood, particularly regarding the potential relationship between these changes and the severity of scoliosis. To date, there have been no reports addressing whether variations in body composition significantly influence the curvilinear progression of AIS. Given that AIS is associated with developmental abnormalities and exhibits a dynamic progression, it is crucial to investigate the changes in body composition throughout the growth and developmental phases, as this may yield novel insights for clinical interventions targeting AIS patients. The objective of our research is to elucidate the changes in body composition of AIS patients during their growth and development by analyzing body composition at various stages of bone maturation. To our knowledge, this study represents the first comprehensive report on the body composition of AIS patients across different bone maturation stages, thereby addressing a notable gap in the existing literature and providing a theoretical foundation for exploring the mechanisms underlying changes in body composition among AIS patients. Through correlation analysis, we aim to establish the relationship between body composition and the severity of scoliosis in this population.

We obtained the whole-body muscle percentage and subcutaneous fat percentage, as well as the local muscle percentage and subcutaneous fat percentage of the patients, using a portable body fat scale. The principle of the body fat scale is bioelectrical impedance analysis (BIA)^21^. Compared with other methods of measuring body composition, such as quantitative computed tomography (qCT) or dual-energy X-ray absorptiometry (DXA), BIA is relatively simple, fast, non-invasive, and easily accessible. It also avoids radiation exposure. Moreover, BIA has a good correlation with DXA in estimating body composition, and therefore, it is widely used in research on body composition^14,22,23^. Hence, we employed it for our study.

## Methods

### Subjects

This study employs a cross-sectional design and is conducted within the population of individuals with AIS. This study was approved by the Ethics Review Committee of Xiangya Hospital of Central South University before data collection and analysis (ethical code, 2022020575). We collected data from 243 AIS patients who visited the Spine Surgery Clinic at Xiangya Hospital, Central South University. This study utilized data from Spine Surgery Clinic at Xiangya Hospital, Central South University, the dates of data access are as follows: 10-01-2023 to 31-8-2024. All study participants provided written informed consent before the evaluation, and all research activities followed the principles of the Helsinki Declaration. Inclusion criteria were as follows: (1) Diagnosed with idiopathic scoliosis with a Cobb angle > 10°, (2) Aged between 10 and 20 years, (3) First-time consultation with no prior medical guidance or treatment, (4) Complete basic information and clinical data. Exclusion criteria included: 1) Congenital, neuromuscular, or other clearly defined causes of scoliosis.2) Other musculoskeletal system disorders, such as muscle atrophy, spinal kyphosis, spinal trauma, or a history of spinal surgery. 3) Prior medical treatment or guidance for AIS patients. 4)There is a history of poor bone development, connective tissue diseases, endocrine diseases and glucocorticoid treatment.

### Data collection

The general information of AIS patients was collected, including gender, age, height, menarche and weight. Body composition measurements were taken using a portable body fat scale (Omron, HBF-701, Japan) of the same brand and type. This body fat scale has four electrode contact points that connect to the limbs of the human body, allowing it to measure muscle percentage and subcutaneous fat percentage at different body parts, including the whole-body, trunk, and limbs.

Measurement method: All patients were measured in a fasting state in the morning, with their bladders emptied beforehand. During the measurement, patients removed their clothing and shoes, and height was measured (to the nearest 0.01 m). The operator entered the patient’s height, age, and gender into the machine, ensuring that the bare limbs were in close contact with the four electrodes of the body fat scale. The instrument automatically measured body weight (to the nearest 0.01 kg) and calculated the whole-body and local muscle percentage and subcutaneous fat percentage using its internal algorithm. The results were accurately recorded by the operator. To minimize operator error, all body composition measurements were performed by the same person.

The main curve Cobb angle was measured from whole-spine posteroanterior (P/A) X-rays taken in a standing position, along with recording the Risser sign on the non-dominant side and the apical vertebra rotation(according to Nash and Moe). The X-ray imaging and body composition measurements were conducted on the same day to ensure the basic consistency of the two data sets.

The Risser sign is closely related to spinal skeletal maturity, and its stages are associated with the degree of closure of the spinal growth plates. It is now widely used in scoliosis research and is an important tool for assessing adolescent skeletal development. To investigate whether there are differences in body composition among AIS patients at different stages of skeletal development, we categorized patients into groups 0–5 based on the Risser sign and compared body composition across these groups. Patients were also divided into three groups based on Cobb angle: mild group (< 20°), moderate group (20°-40°), and severe group (≥ 40°). To eliminate the impact of individual differences, we corrected the patients’ body composition using body mass index (BMI) and conducted data analysis on the correcred body composition. To clarify the correlation between body composition and the severity of scoliosis, we performed correlation analyses between body composition and Cobb angle as well as the AVR. All imaging parameters were measured independently by two physicians, and the mean values were calculated for subsequent statistical analysis.

### Statistical analysis

Continuous variables were described as means and standard deviations (x̅ ± SD). All data were tested for normality before statistical analysis. Differences among the three groups were analyzed using one-way analysis of variance (ANOVA), and homogeneity of variance was tested before performing ANOVA. If variances were homogenous, the LSD test was used; if not, the Tamhane test was employed. The differences in composition ratios were analyzed using the chi-square test. The correlation between muscle percentage and Cobb angle and AVR was analyzed using the Spearman correlation coefficient.The differences between two groups were analyzed using independent sample t-tests. Statistical analysis was performed using the Statistical Package for the Social Sciences (SPSS software, version 25.0, Chicago, USA). Statistical significance was set at *p* < 0.05.

## Results

### Analysis of whole-body and local body composition in AIS patients with different risser sign stages

**Fig. 1:**
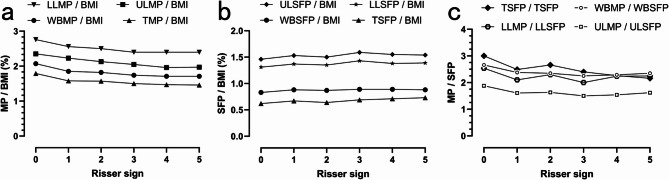
(**a**) Whole-body and local muscle percentage corrected by BMI in AIS patients at different stages of skeletal development; (**b**) Whole-body and local subcutaneous fat percentage corrected by BMI in AIS patients at different stages of skeletal development; (**c**) Ratio of muscle percentage to subcutaneous fat percentage in whole-body and local areas of AIS patients at different stages of skeletal development.

This study included 243 AIS patients, comprising 51 males and 192 females, aged 10 to 20 years. Among the cohort, there were 22 patients exhibiting Risser’s sign at grade 0, 19 at grade 1, 37 at grade 2, 61 at grade 3, 82 at grade 4, and 22 at grade 5. Of the 192 female patients, 140 had experienced menarche, while 52 had not. We classified AIS patients into groups based on Risser sign stages (0–5). There were significant statistical differences in age, height, weight, and BMI among AIS patients with different Risser signs (*p* < 0.05). As the Risser sign stage increased, age, height, weight, and BMI also gradually increased. After being adjusted by BMI, there were significant statistical differences in whole-body muscle percentage, trunk muscle percentage, and limb muscle percentage among the groups (*p* < 0.05), indicating that as the Risser sign stage increased, the muscle percentages in the whole-body, trunk, and limbs gradually decreased. However, there were no significant statistical differences in whole-body subcutaneous fat percentage, trunk subcutaneous fat percentage, and limb subcutaneous fat percentage among the groups(*p* > 0.05).(Table 1; Fig. 1).

**Table 1:** Comparison among different grades of Risser sign.

Group	0	1	2	3	4	5	F	*P*
Gender (male/female)	7/15	4/15	6/31	11/50	18/64	5/17		0.781
Menarche(Yes/No)	5/10	5/10	16/15	39/11	59/5	16/1		< 0.001^***^
Age	11.77 ± 1.38	12.11 ± 1.45	13.03 ± 1.38	14.10 ± 1.49	16.23 ± 2.07	17.77 ± 2.20	54.699	< 0.001^***^
Hight (m)	1.53 ± 0.11	1.56 ± 0.11	1.58 ± 0.09	1.61 ± 0.07	1.64 ± 0.08	1.65 ± 0.08	8.955	< 0.001^***^
Weight(kg)	37.86 ± 8.89	41.71 ± 8.38	45.02 ± 9.47	48.14 ± 8.51	50.50 ± 7.03	51.67 ± 9.09	11.816	< 0.001^***^
BMI	16.13 ± 2.15	17.68 ± 2.72	18.16 ± 2.35	18.37 ± 2.56	18.78 ± 2.02	19.04 ± 3.20	4.953	< 0.001^***^
Cobb angle ( °)	36.67 ± 23.48	37.21 ± 15.60	34.80 ± 18.71	36.51 ± 16.50	36.41 ± 14.22	31.46 ± 13.80	0.411	0.841
WBSFP / BMI (%)	0.83 ± 0.18	0.88 ± 0.21	0.87 ± 0.20	0.89 ± 0.23	0.89 ± 0.25	0.88 ± 0.28	0.273	0.928
WBMP / BMI (%)	2.07 ± 0.37	1.85 ± 0.39	1.82 ± 0.37	1.74 ± 0.33	1.71 ± 0.35	1.71 ± 0.43	3.975	0.002^**^
ULSFP / BMI (%)	1.46 ± 0.44	1.53 ± 0.37	1.50 ± 0.43	1.59 ± 0.43	1.55 ± 0.46	1.54 ± 0.52	0.349	0.883
ULMP / BMI (%)	2.35 ± 0.68	2.23 ± 0.50	2.13 ± 0.49	2.05 ± 0.43	1.96 ± 0.39	1.97 ± 0.53	3.251	0.007^**^
TSFP / BMI (%)	0.62 ± 0.15	0.67 ± 0.16	0.64 ± 0.16	0.69 ± 0.18	0.71 ± 0.19	0.73 ± 0.22	1.548	0.176
TMP / BMI (%)	1.79 ± 0.34	1.58 ± 0.34	1.57 ± 0.35	1.50 ± 0.31	1.47 ± 0.39	1.46 ± 0.42	3.250	0.007^**^
LLSFP / BMI (%)	1.31 ± 0.38	1.37 ± 0.33	1.35 ± 0.38	1.43 ± 0.38	1.38 ± 0.43	1.39 ± 0.45	0.350	0.882
LLMP / BMI (%)	2.76 ± 0.67	2.56 ± 0.54	2.50 ± 0.50	2.40 ± 0.48	2.40 ± 0.46	2.40 ± 0.57	3.706	0.026^**^
WBMP / WBSFP	2.66 ± 1.06	2.38 ± 1.41	2.35 ± 1.31	2.25 ± 1.28	2.29 ± 1.56	2.35 ± 1.70	0.285	0.921
ULMP / ULSFP	1.88 ± 1.17	1.61 ± 0.78	1.63 ± 1.01	1.50 ± 0.83	1.54 ± 1.05	1.62 ± 1.17	0.511	0.768
TSFP / TSFP	3.00 ± 1.17	2.49 ± 1.01	2.66 ± 1.23	2.40 ± 1.11	2.26 ± 1.26	2.16 ± 1.16	1.691	0.138
LLMP / LLSFP	2.54 ± 1.82	2.10 ± 1.15	2.30 ± 1.71	2.00 ± 1.21	2.24 ± 1.80	2.25 ± 1.83	0.436	0.823

The data are expressed as: x̅±SD; abbreviations: BMI: body mass index; WBMP: Whole-body muscle percentage; TMP: trunk muscle percentage; WBSFP: Whole-body subcutaneous fat percentage, TSFP: trunk subcutaneous fat percentage; ULMP upper limb muscle percentage; ULSFP: upper limb subcutaneous fat percentage; LLMP: lower limb muscle percentage; ULSFP: upper limb subcutaneous fat percentage. * *p* < 0.05, ** *p* < 0.01, *** *p* < 0.001.

### Comparison of whole-body and local body composition in AIS patients with different severity levels

Patients were divided into three groups based on the size of the Cobb angle: mild group(< 20°), moderate group(22°-40°), and severe group(≥ 40°). There were 29 patients in the mild group, 136 in the moderate group, and 78 in the severe group. The gender ratios, age, height, weight, BMI, and Risser sign among the three groups showed no statistical differences (*p* > 0.05). After being adjusted by BMI, there were significant statistical differences in muscle percentage and subcutaneous fat percentage in the whole-body, trunk, and limbs among the three groups (*p* < 0.05). Patients in the mild group had significantly higher muscle percentage in both the whole-body and locally compared to those in the moderate and severe groups, while their subcutaneous fat percentages were significantly lower than those in the moderate and severe groups. The ratios of muscle percentage to subcutaneous fat percentage in the whole-body, trunk, and limbs also showed significant statistical differences (*p* < 0.05), with the mild group exhibiting significantly higher ratios than the moderate and severe groups. Additionally, there were significant statistical differences in the AVR among the three groups (*p* < 0.05), with the mild group showing the least rotation and the severe group the most (Table 2).

**Table 2:** Comparison of three groups of patients.

group	mild group(<20°)	moderate group(20°- 40°)	severe group(≥40°)	F	*P*
Gender (male/female)	7/22	23/113	21/57		0.203
Menarche (Yes/No)	16/6	83/30	41/16		0.978
Height (m)	1.60 ± 0.08	1.62 ± 0.09	1.59 ± 0.10	2.252	0.107
Age	13.97 ± 2.38	14.74 ± 2.52	14.67 ± 2.65	1.111	0.331
Weight (kg)	44.72 ± 10.74	48.39 ± 8.58	46.49 ± 9.34	2.452	0.088
BMI (kg/m^2^)	17.45 ± 2.61	18.47 ± 2.40	18.26 ± 2.60	2.037	0.133
Risser sign	2.66 ± 1.56	3.01 ± 1.36	2.91 ± 1.43	0.800	0.450
Cobb angle ( °)	15.31 ± 2.86	29.63 ± 5.74	54.27 ± 14.93	241.547	< 0.001^***^
RAV	0.45 ± 0.63	1.21 ± 0.90	2.17 ± 1.11	42.827	< 0.001^***^
WBSFP / BMI (%)	0.77 ± 0.25	0.90 ± 0.21	0.89 ± 0.24	4.044	0.019^*^
WBMP / BMI (%)	1.96 ± 0.34	1.74 ± 0.37	1.77 ± 0.36	4.287	0.015^*^
ULSFP / BMI (%)	1.36 ± 0.49	1.57 ± 0.41	1.55 ± 0.47	3.065	0.048^*^
ULMP / BMI (%)	2.33 ± 0.46	2.02 ± 0.46	2.04 ± 0.48	5.349	0.005^**^
TSFP / BMI (%)	0.59 ± 0.19	0.70 ± 0.17	0.69 ± 0.18	3.986	0.020^*^
TMP / BMI (%)	1.68 ± 0.30	1.50 ± 0.39	1.52 ± 0.34	3.045	0.049^*^
LLSFP / BMI (%)	1.18 ± 0.46	1.42 ± 0.37	1.39 ± 0.41	4.180	0.016^*^
LLMP / BMI (%)	2.74 ± 0.51	2.41 ± 0.50	2.44 ± 0.52	5.135	0.007^**^
WBMP / WBSFP	2.94 ± 1.38	2.21 ± 1.36	2.33 ± 1.44	3.283	0.039^*^
ULMP / ULSFP	2.02 ± 0.94	1.50 ± 0.96	1.58 ± 1.03	3.338	0.037^*^
TSFP / TSFP	3.09 ± 1.22	2.30 ± 1.15	2.40 ± 1.19	5.005	0.007^**^
LLMP / LLSFP	2.92 ± 1.79	2.07 ± 1.55	2.17 ± 1.58	3.401	0.035^*^

The data are expressed as: x̅±SD; abbreviations: BMI: body mass index; WBMP: Whole-body muscle percentage; TMP: trunk muscle percentage; WBSFP: Whole-body subcutaneous fat percentage, TSFP: trunk subcutaneous fat percentage; ULMP upper limb muscle percentage; ULSFP: upper limb subcutaneous fat percentage; LLMP: lower limb muscle percentage; ULSFP: upper limb subcutaneous fat percentage. RAV: the rotation of the apical vertebra. * *p* < 0.05, ** *p* < 0.01, *** *p* < 0.001.

### Correlation analysis of whole-body and trunk muscle percentage and subcutaneous fat percentage with cobb angle

**Fig. 2:**
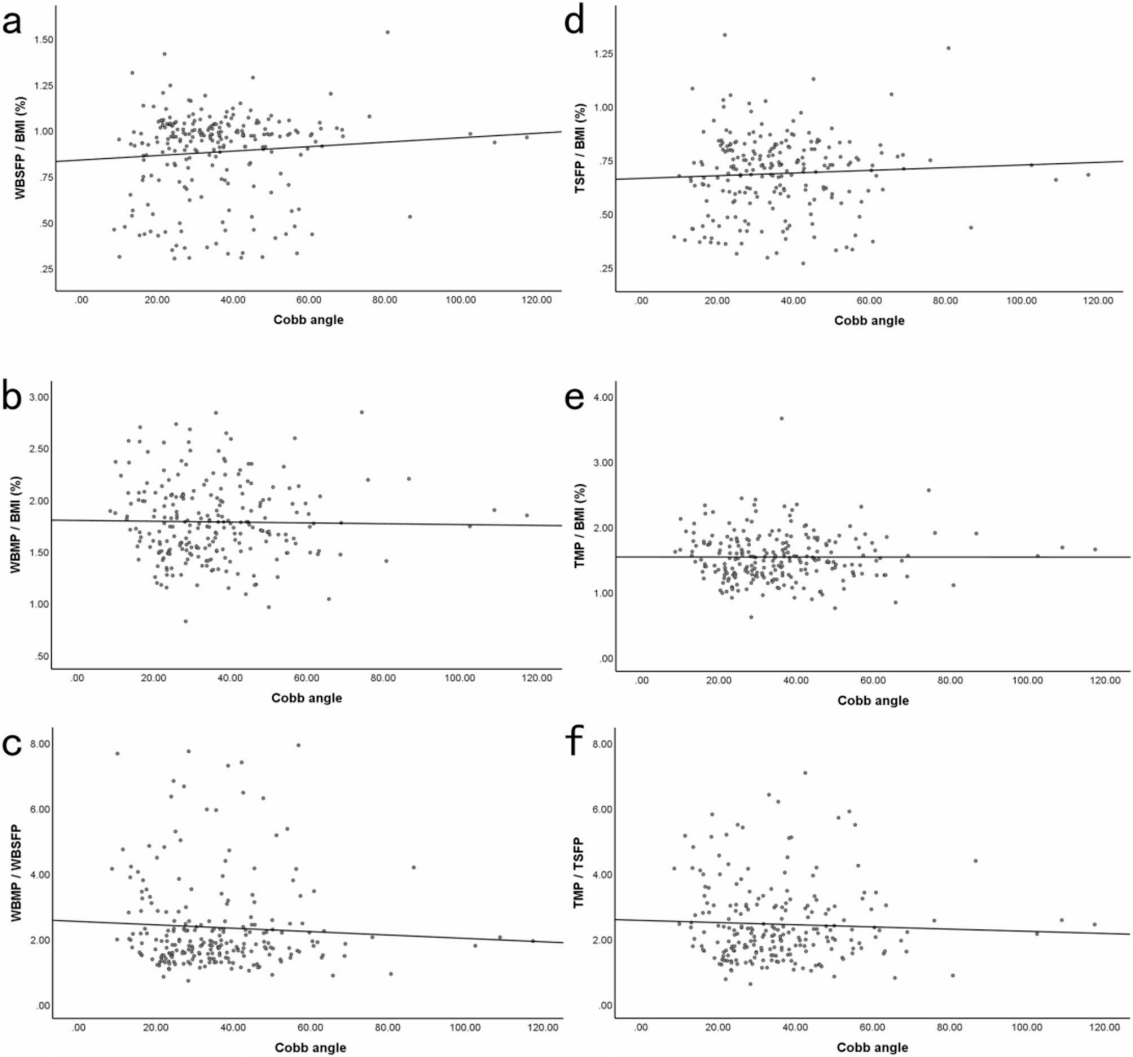
(**a**) Correlation between whole-body subcutaneous fat percentage corrected by BMI and Cobb angle; (**b**) Correlation between whole-body muscle percentage corrected by BMI and Cobb angle; (**c**) Correlation between the ratio of whole-body muscle percentage to whole-body subcutaneous fat percentage and Cobb angle; (**d**) Correlation between trunk subcutaneous fat percentage corrected by BMI and Cobb angle; (**e**) Correlation between trunk muscle percentage corrected by BMI and Cobb angle; (**f**) Correlation between the ratio of trunk muscle percentage to trunk subcutaneous fat percentage and Cobb angle.

The whole-body and trunk muscle percentage showed a weak negative correlation with the Cobb angle, with no statistical significance (*p* > 0.05). The whole-body and trunk subcutaneous fat percentage exhibited a weak positive correlation with the Cobb angle, also with no statistical significance (*p* > 0.05). Additionally, the ratio of muscle percentage to subcutaneous fat percentage in the whole-body and trunk showed a weak negative correlation with the Cobb angle, with no statistical significance (*p* > 0.05) (Table 3; Fig. 2).

**Table 3:** Correlation analysis between whole-body and trunk muscle percentage and Cobb angle.

Test	Correlation coefficients	*P* value
WBMP / BMI(%)	− 0.024	0.707
WBSFP / BMI(%)	0.071	0.279
WBMP / WBSFP	− 0.047	0.478
TMP / BMI(%)	− 0.011	0.868
TSFP / BMI(%)	0.051	0.445
TMP / TSFP	− 0.038	0.568

Abbreviations: WBMP: Whole-body muscle percentage; TMP: trunk muscle percentage

WBSFP: Whole-body subcutaneous fat percentage, TSFP: trunk subcutaneous fat percentage

### Correlation analysis of whole-body and trunk muscle percentage and subcutaneous fat percentage with AVR

The whole-body muscle percentage showed a weak negative correlation with AVR, with statistical significance (*p* < 0.05). The whole-body subcutaneous fat percentage exhibited a weak positive correlation with AVR, also with statistical significance (*p* < 0.05). Additionally, the ratio of whole-body muscle percentage to subcutaneous fat percentage showed a weak negative correlation with AVR, with statistical significance (*p* < 0.05). The trunk muscle percentage demonstrated a weak negative correlation with AVR, but this difference was not statistical significance (*p* > 0.05). The trunk subcutaneous fat percentage showed a weak positive correlation with AVR, again with no statistical significance (*p* > 0.05). Finally, the ratio of trunk muscle percentage to subcutaneous fat percentage exhibited a weak negative correlation with apical vertebra rotation, with no statistical significance (*p* > 0.05) (Table 4).

**Table 4:** Correlation analysis between whole-body and trunk muscle percentage and RAV.

Test	Correlation coefficients	*P* value
WBMP / BMI(%)	− 0.131	0.042^*^
WBSFP / BMI(%)	0.150	0.021^*^
WBMP / WBSFP	− 0.169	0.009^**^
TMP / BMI(%)	− 0.109	0.090
TSFP / BMI(%)	0.112	0.095
TMP / TSFP	− 0.131	0.050^*^

Abbreviations: WBMP: Whole-body muscle percentage; TMP: trunk muscle percentage

WBSFP: Whole-body subcutaneous fat percentage, TSFP: trunk subcutaneous fat percentage. * *p* < 0.05, ** *p* < 0.01, *** *p* < 0.001

## Discussion

Severe scoliosis not only affects the aesthetic appearance of the body but can also impair cardiopulmonary function. Early management of scoliosis can help slow its progression. Muscle and bone are two fundamental elements of the musculoskeletal system, closely linked and mutually regulating each other. Muscle tissue is a crucial protective factor against osteoporosis; it can modulate bone mass through mechanical loading and regulate bone metabolism by secreting factors such as myostatin and osteocalcin^15,24^ A decline in mechanical loading due to muscle atrophy can trigger bone loss^25^. Muscle and adipose tissues are important components of the human body, and their ratio can reflect an individual’s basal metabolic rate. There is also a complex relationship between adipose tissue and bone mass^26^. As an abnormal developmental condition, AIS may have certain associations with muscle and adipose tissues. Therefore, it is essential to clarify the changes in muscle and adipose tissues during the developmental process of patients with AIS.

The Risser sign is an important indicator for assessing skeletal maturity, beginning in puberty and reaching its maximum grade at the end of the growth period, it is widely used in research on AIS to categorize different stages of growth and development in AIS patients^27,28^. To explore body composition at various developmental stages during puberty in AIS patients, we divided the patients into six groups based on Risser sign grade 0–5. The results indicated that as age increases, the skeletal maturity of AIS patients also increases, which is part of the normal skeletal development process. Additionally, as skeletal maturity improves, the physical parameters (height, weight, BMI) of AIS patients gradually increase. No statistical differences were found in the Cobb angle among the six groups, suggesting that the rapid progression during the peak growth phase is specific to individual patients rather than a phenomenon of the entire AIS population. Because the Risser sign grade 0–2 represent the period of greatest growth potential and fastest development in adolescents, yet the Cobb angles of these patients do not exceed those of other groups. As the Risser sign grade and age increase, there is a gradual rise in the number of individuals experiencing menarche, as demonstrated in the study conducted by Neal et al.^29^. Interestingly, our study found that as skeletal maturity and body weight increased, the muscle percentage in the whole-body, trunk, and limbs of AIS patients showed a decreasing trend, stabilizing at Risser sign grade 4–5. The ratio of whole-body and local muscle percentage to subcutaneous fat percentage was greatest at Risser sign grade 0, while whole-body and local subcutaneous fat percentage were lowest at this grade, fluctuating slightly from grade 1–5. It is known that during normal adolescent development, puberty is a critical phase for muscle development; skeletal muscle mass increases with age, initially rising and then gradually declining as a percentage of body weight^30,31^. Age-related muscle loss can begin as early as after age 30 and accelerates after age 60^32^. A study on the skeletal muscle development characteristics of normal children and adolescents in the UK showed that limb muscle mass in boys aged 5–16 and girls aged 5–17 consistently increased^30^. We also found that with increasing skeletal maturity, the whole-body and local subcutaneous fat percentage in AIS patients shows an increasing trend, which is largely consistent with reports of increased fat mass in girls during late puberty^31^.

Our research findings indicate that AIS patients experience an early decline in muscle percentage during puberty, while the increase in adipose tissue is not significant, representing a novel discovery. Muscle growth is influenced by various factors, including genetics, nutrition, physical activity levels, and hormonal levels^33–35^. The reasons for muscle growth during puberty include the increased secretion of sex hormones (androgens and estrogens). Testosterone promotes skeletal muscle protein synthesis, enhancing muscle mass and strength^36^; estrogen may indirectly promote muscle strength and energy by increasing central nervous system excitability, and it may also directly influence muscle growth by affecting protein synthesis and muscle differentiation^37^. Additionally, an increase in body weight provides the necessary stimulus for muscle growth; as body weight increases, the direct loading on muscles also rises, further stimulating muscle growth^38^. Studies have shown that AIS patients have lower levels of androgens and serum estradiol compared to normal individuals^39^, Leptin has been shown to enhance muscle mass by inhibiting the degradation of troponin and promoting the proliferation of muscle cells; however, individuals with AIS exhibit low levels of leptin, accompanied by reactive changes^11,40^. In patients with AIS, alterations in the expression of muscle-related genes and muscle atrophy are observed in the paravertebral muscles on the concave side of the spinal curve. Specifically, the paravertebral muscles of AIS patients demonstrate a reduced proportion of type I muscle fibers, an increased proportion of type II muscle fibers, greater muscle thickness, and notable fat infiltration^41^ on the concave side of the curve. Furthermore, research conducted by Dai et al.^42^ indicates that the sensitivity of AIS muscles to glucose and insulin is diminished. Collectively, these changes may contribute to a lower rate of muscle increase relative to other body components during the progression of AIS, ultimately resulting in a decreased muscle percentage. The previous results lead us to suspect that there is a phenomenon of sustained decline in sex hormones within AIS patients, which gradually stabilizes during the later stages of skeletal development, this hypothesis requires further research for confirmation. Our research findings suggest that as bone maturity progresses, both the height and weight of patients with AIS increase. However, the alterations in the percentage of subcutaneous fat, both systemically and locally, are not statistically significant. This observation implies that during the progression of AIS, the distribution of subcutaneous fat does not exhibit substantial changes, which may be influenced by hormonal factors. Previous studies have indicated that, in comparison to individuals without AIS, there are notable variations in the levels of leptin and adiponectin among AIS patients^11,43^. The ratio of whole-body and local muscle percentage to subcutaneous fat percentage is greatest at Risser sign grade 0, while whole-body and local subcutaneous fat percentages are lowest at this grade, which aligns with the typical development of subcutaneous fat during puberty in the normal population, where subcutaneous fat initially increases and then stabilizes. Although sex hormones also influence changes in subcutaneous fat percentage^44,45^ ,our results suggest that in the AIS population, the impact of sex hormones on muscle development may be greater than their effect on subcutaneous fat. In conclusion, the alterations in body composition observed in the aforementioned patients with AIS may be associated with hormonal factors, genetic predispositions, and muscle atrophy.

To investigate whether there is an association between body composition and the severity of scoliosis in AIS patients, we divided all patients into three groups based on Cobb angle: mild, moderate, and severe. There were no differences among the three groups in terms of age, height, weight, BMI, Risser sign, and Menarche indicating that the severity of scoliosis is unrelated to these factors, which seems to be inconsistent with some studies. A prospective study indicated that a lower BMI and weight at age 10 are associated with the occurrence of scoliosis at age 15^10^. Research by Wang et al.^6^ showed a negative correlation between Cobb angle and weight, and a positive correlation with age. Other studies have also found that patients in the severe AIS group tend to have higher weight and BMI^14^. In a study of 210 Japanese adolescent idiopathic scoliosis (AIS) girls, Miyagi et al.^46^ demonstrated that the body mass index (BMI), lean muscle mass index (LMI), and estimated bone mass index (eBoneMI) were significantly lower in the moderate scoliosis group than in the severe scoliosis group. Our research findings suggest that there is no significant difference in the menarche status among patients with varying degrees of scoliosis. While menarche is a critical milestone for understanding the progression of AIS patients^47^, it may offer advantages for individual cases. In the AIS population, the magnitude of the scoliosis angle does not appear to be influenced by the presence or absence of menarche. Furthermore, these indices exhibited weak but significant correlations with the Cobb angle. The discrepancies in results may relate to factors such as ethnicity, sample size, and sampling error. Therefore, further multicenter studies with larger sample sizes are needed to clarify the relationship between age, weight, BMI, and scoliosis severity. Our findings reveal that the whole-body and local muscle percentages in the mild group are higher than those in the moderate to severe groups, while the whole-body and local subcutaneous fat percentages in the mild group are lower than those in the moderate to severe groups, which is largely consistent with the findings of Edyta Matusik et al.^14^. In our study, patients with larger Cobb angles exhibited greater AVR. Previous research has indicated that AVR is an important factor increasing the risk of curve progression^48^. We conducted correlation analyses between whole-body and trunk muscle percentage and subcutaneous fat percentage with the Cobb angle and AVR, finding a weak negative correlation between whole-body muscle percentage and AVR, and a weak positive correlation between whole-body subcutaneous fat percentage and AVR. Although the correlations observed are relatively weak, they may still possess clinical significance. These correlations indicate that, in clinical practice, an increase in the percentage of whole-body muscle and a reduction in the percentage of subcutaneous fat may contribute to a decrease in the degrees of AVR and Cobb angles. Furthermore, it is plausible that this weak correlation could be strengthened in studies with larger sample sizes, which necessitates further investigation. However, the underlying mechanisms driving this correlation remain unclear. It is possible that an increase in muscle percentage enhances spinal stability.

In this study, we revealed the developmental patterns of whole-body and local body composition during puberty in the AIS population by comparing muscle percentage and subcutaneous fat percentage across different stages of skeletal development. Our findings showed that muscle percentage in the mild group of scoliosis patients was significantly higher than that in the moderate to severe groups, while subcutaneous fat percentage was lower in the mild group. Correlation analyses indicated a weak positive correlation between whole-body muscle percentage and AVR, and a weak negative correlation between whole-body subcutaneous fat percentage and AVR. Therefore, we propose that the reduction in whole-body muscle percentage and the increase in subcutaneous fat percentage are risk factors for curve progression in AIS patients. Enhancing whole-body muscle percentage in AIS patients may help reduce the risk of scoliosis progression, with the optimal intervention period being when the Risser sign is grade 0–1, as muscle percentage declines most rapidly during this timeframe.

There are several limitations to our study. It is a single-center, cross-sectional study, and the population is derived from central and southern regions of China, resulting in a limited sample size, particularly with a relatively small number of patients in the mild group, this may be related to the lower detection and treatment rates for such patients due to the less pronounced deformity. The smaller sample size in the mild group may restrict the statistical power to detect differences within this subgroup. Our research involved a cross-sectional comparison of body composition among patients at different stages of skeletal development within the AIS population, and we did not conduct a full-cycle follow-up of individual patients to collect their body composition data at various developmental stages, because obtaining complete follow-up data for a patient throughout their entire adolescent developmental period is challenging. Individual genetic differences and baseline physical health conditions cannot be eliminated among participants, and these variations may potentially influence the study outcomes. In the future, it will be necessary to conduct multi-center studies with large sample sizes or longitudinal cohort studies to validate our findings, address the limitations of current research, and facilitate further exploration in this field. Nevertheless, this study is the first to report the dynamic developmental patterns of body composition in AIS patients and the first to establish a correlation between muscle percentage and subcutaneous fat percentage and scoliosis severity in this population. The findings of our research may identify potential intervention targets for the treatment of AIS. Clinically, interventions aimed at increasing muscle mass in patients while simultaneously reducing subcutaneous fat may prove effective. Furthermore, enhanced therapeutic outcomes may be attainable through the use of combined braces or alternative treatment modalities. Nevertheless, further research is necessary to validate these hypotheses.

## Conclusion

In AIS patients, as skeletal maturity progresses, there is a gradual decrease in both whole-body and local muscle percentage, and the ratio of muscle to fat percentage tends to decline. In the context of clinical intervention, a range of strategies aimed at enhancing muscle mass and decreasing subcutaneous fat percentage—such as increasing physical activity, modifying dietary intake, and altering lifestyle habits—may contribute to the conservative management of AIS. The optimal time to intervene for reduced muscle percentage in AIS patients is during Risser sign stage 0–1.

## Data Availability

The datasets used and analysed during the current study are available to be collected from the corresponding author on reasonable request.
